# Dynamic changes in bacterial communities in the recirculating nutrient solution of cucumber plug seedlings cultivated in an ebb-and-flow subirrigation system

**DOI:** 10.1371/journal.pone.0232446

**Published:** 2020-04-30

**Authors:** Chun-Juan Dong, Qian Li, Ling-Ling Wang, Qing-Mao Shang

**Affiliations:** Key Laboratory of Horticultural Crop Biology and Germplasm Innovation, Institute of Vegetables and Flowers, Chinese Academy of Agricultural Sciences, Ministry of Agriculture, Beijing, People’s Republic of China; Osmania University, INDIA

## Abstract

Ebb-and-flow subirrigation systems are highly efficient, water-saving and environmentally friendly. However, one concern with these recirculating systems is the possible transmission of plant pathogens. Here, through *16S rRNA*-targeted Illumina sequencing, the bacterial dynamics in a recirculating nutrient solution were characterized for cucumber plug seedlings cultivated in an ebb-and-flow system in summer and winter. Both the bacterial number and diversity in the nutrient solution increased immediately after the first irrigation cycle; then, these values were gradually stable with recirculating irrigation. In summer and winter, different bacterial compositions and changing patterns were observed. In summer, the predominant genera in the nutrient solution included *Comamonas*, *Pseudomonas*, *Acinetobacter*, *Reyranella*, *Sphingobium*, *Bradyrhizobium*, *Sphingomonas*, and *Acidovorax*. Of those genera, during recirculating irrigation, the relative abundance of *Bradyrhizobium* gradually decreased, whereas those of *Pseudomonas*, *Reyranella*, *Sphingobium*, *Sphingomonas*, and *Acidovorax* gradually increased. In winter, the bacterial communities were mainly composed of *Nevskia*, *Bosea*, *Sphingobium*, *Acidovorax*, *Pseudomonas*, and *Hydrocarboniphaga*. Of those genera, the relative abundance of *Bosea*, *Sphingobium*, and *Acidovorax* showed an increasing trend, whereas those of *Nevskia* and *Hydrocarboniphaga* decreased overall. Furthermore, in both summer and winter, no plant pathogenic bacteria on cucumber could be detected; however, some potentially beneficial bacteria, including *Comamonas testosteroni*, *Acinetobacter baumannii*, *Pseudomonas aeruginosa*, *P*. *koreensis* and *Sphingobium yanoikuyae*, colonized the nutrient solution and exhibited increased relative abundances during irrigation. The colonization of these bacteria might facilitate the plant growth promotion. Inoculation of the microbes from the effluent nutrient solution also promoted the growth of cucumber seedlings, but did not lead to any disease. The present data elucidate the bacterial dynamics in a cucumber cultivation ebb-and-flow system and provide useful information for biological control during cucumber seedling production.

## Introduction

Ebb-and-flow irrigation is a highly efficient, water-saving and environmentally friendly subirrigation technique that is emerging to replace traditional overhead irrigation systems in nursery plant production. Ebb-and-flow systems use an automatic flood and drain watering technique, in which plants are flooded temporarily and periodically [1]. The water or nutrient solution in the reservoir ascends to the growth tray via a timer-activated water pump, accumulates to a certain level, and remains for a set amount of time, providing water and nutrients to the plants. After a predetermined time, the solution is drained back into the reservoir through a tubing system [2–4]. The drainage water from ebb-and-flow systems is usually recycled to save water and nutrients, thereby preventing fertilizers and pesticides from entering surface and groundwater [5,6]. Ebb-and-flow systems can also reduce labor costs by eliminating the need for hand-watering and reducing the incidence of foliar diseases by keeping the foliage dry [2,7]. As a result of these benefits, the supply of and demand for ebb-and-flow systems have dramatically increased in horticultural production [7], especially in the intensive cultivation of plug seedlings.

Despite the numerous benefits, one concern with recirculating ebb-and-flow systems is the possible transmission of plant pathogens [8]. The nutrient solution can be colonized by various microorganisms, including many plant pathogens. First, the nutrient solution is inhabited by a large number of indigenous microorganisms and can also be contaminated by various microorganisms from different sources, including air, seeds, plants, substrates, and personnel [9]. Furthermore, almost all ebb-and-flow systems are located indoors in greenhouses, where the environmental parameters are all set to optimize plant growth and minimize plant stress [3,10]. Of course, the microorganisms in the nutrient solution are also exposed to this narrowly defined environment, which supports microbial growth and production. Once the microbes colonize the nutrient solution, they can be dispersed rapidly throughout an entire crop by recirculated irrigation [11,12]. The spread of microorganisms in recirculating systems that use neither soil nor organic media in hydroponic cultivation systems has been well documented [13–16]. Some common water-borne plant pathogens, such as *Xanthomonas*, *Fusarium*, *Phytophthora*, and *Pythium*, have been detected in ebb-and-flow systems [5,17–19]. However, little is known about the microbial dissemination in the ebb-and-flow irrigation systems.

To avoid the spread of pathogens in closed ebb-and-flow irrigation systems, disinfection of the recycled nutrient solution after each irrigation cycle is standard practice [6, 20]. The use of heating, ozone or UV-radiation techniques to control pathogen dissemination for nutrient solutions after each irrigation cycle is now used commercially by greenhouse growers, especially in China. These active methods are often accompanied by an initial filtering of the solutions through slow sand filtration to remove large pieces of plant or other debris [6, 15]. However, these disinfection methods often require an excessively large investment [21]. Additionally, these methods eliminate most of the nonpathogenic microflora, most of which are beneficial to plants or, at least, do not cause significant repression of plant growth [2]. Whether the disinfection of nutrient solution after each irrigation cycle is essential in a recirculating ebb-and-flow system needs to be evaluated. If no disinfection process is used, the remaining beneficial microbes in the solutions could contribute to the promotion of plant growth. Analysis of the dynamic changes in the microbial community in recirculating ebb-and-flow systems, especially depicting the distribution patterns of the beneficial and pathogenic microbes, will be needed to develop a more rational and economic strategy for the disinfection of recirculating nutrient solution.

Cucumber (*Cucumis sativus* L.) is an economically important cash crop. To improve the quality and yield, cucumber is commonly cultivated by the transplantation of plug seedlings [22], and ebb-and-flow systems have been widely used for the commercial production of cucumber plug seedlings. Many bacterial diseases are distributed worldwide and cause heavy economic losses under favorable climatic conditions by decreasing the quality of cucumber plug seedlings [23]. Unfortunately, reports on the bacterial community in recirculating ebb-and-flow systems for cucumber seedling cultivation are sparse. In the present study, through Illumina MiSeq sequencing technology, the dynamic changes in bacterial communities were monitored in the recirculating nutrient solution and substrate within an ebb-and-flow system in both summer and winter. In addition, the changes in the relative abundances of beneficial and pathogenic bacteria were analyzed. This work will provide a comprehensive understanding of the bacterial dynamics in an ebb-and-flow system, which is useful to develop a rational disinfection strategy for recirculating nutrient solutions and will provide some useful bacterial candidates for biological control in cucumber seedling cultivation.

## Materials and methods

### Cultivation of cucumber plug seedlings

Cucumber (*C*. *sativus* L. “Zhongnong 18”) seeds were immersed in deionized water for 6 h at room temperature, disinfected in a 5% NaClO solution for 10 min, washed with sterile water five times, and germinated at a constant temperature of 28°C for 24 h. Then, seeds with 2–3 mm radicles were selected and sown in a 72-well plug tray containing substrate (peat: vermiculite: perlite = 3:1:1, v/v) at a depth of 1.0 cm. Each plug hole contained one seed and was covered with vermiculite after sowing.

A simplified ebb-and-flow system was designed for this experiment (S1 Fig), including a small growth tray (1 515 × 560 × 40 mm, L×W×H), a submersible pump (85 W, and 3000 L/h), and a 100-L solution reservoir. Water-soluble “20-10-20” fertilizer (Wintong, Shanghai) containing 20% N, 10% P_2_O_5_, and 20% K_2_O was also used for irrigation. The fertilizers were dissolved in deionized tap water to the indicated concentration (Table 1) and used as nutrient solution. During each irrigation, the influent nutrient solution was pumped from the reservoir into the seedling plates to a height of 2 cm and then maintained for 10 min. Subsequently, the effluent nutrient solution was collected back into the reservoir. During the next irrigation, with the addition of a defined volume of water and amount of fertilizer, the nutrient solution was reused as the influent for irrigation. Dynamic changes in the electric conductivity (EC) and dissolved oxygen (DO) content in the nutrient solution during the seedling cultivation period were recorded by a conductivity meter (DDSJ-308A) and portable DO meter (JPBJ-608, Leici, Shanghai, China), respectively (S2 Fig). The cucumber seedlings were grown to the two-leaf stage, which was the normal plant size for transplanting. During the seedling period, six irrigation cycles were performed in both summer and winter (Table 1). Three biological replicates were included in the experiment. The plug trays, ebb-and-flow growth trays, and nutrient solution reservoirs in this study were disinfected with 2% NaClO solution for 30 min and washed with sterile water three times before use.

**Table 1:** The irrigation frequency and fertilizer concentrations of cucumber plug seedlings cultivated in an ebb-and-flow system in summer and winter.

Irrigation No.	Season: summer		Season: winter	
	Irrigation date (dps*)	Fertilizer concentration (mg/L)	Irrigation date (dps*)	Fertilizer concentration (mg/L)
1	4	250	9	250
2	7	250	16	250
3	9	500	21	500
4	14	750	28	750
5	17	1 000	35	1 000
6	20	1 000	41	1 000

* dps, days post sowing.

The experiments were performed in a greenhouse at the Institute of Vegetables and Flowers from June 21 to July 12, 2018 (summer) and from November 15 to December 27, 2018 (winter). The temperature was reduced in summer using a sunshade net with a fan and wet curtain system, while in winter, the temperature was increased using a heater. The environmental parameters in the greenhouse during the seedling cultivation period were monitored in real time with a “Wenshiwawa” system (GIS-4-CE-3V, NERCITA, Beijing) (S3 Fig).

### Counting of the culturable bacteria in the recirculating nutrient solution and substrate samples

The influent and effluent nutrient solutions from each irrigation cycle were collected and filtered through three layers of sterile gauze to remove the plant and other debris. Culturable bacteria for each nutrient solution sample were counted by the dilution plate method. After incubation at 28°C for 24 h, colony-forming units (CFUs) of culturable bacteria were estimated using nutrient agar medium (NA; 1% peptone, 0.3% beef extract, 0.5% NaCl, 1.5% agar, pH 7.2) and expressed as averages of five replicate plates for each duplicate sample with 95% confidence intervals [24].

The substrate samples were collected before sowing (as the initial sample) and at 22 dps (days post sowing) and 43 dps in summer and winter, respectively (as the final samples). At these timepoints, 25 cucumber plug seedlings were randomly selected, and the root substrates were collected. After being evenly mixed, 5 g of the substrates was collected and placed in a flask containing 50 mL of sterile water. After incubating at 28°C with shaking at 160 rpm for 30 min, the extraction solution was collected and filtered through three layers of gauze to remove the substrate debris. The number of culturable bacteria in the extraction solutions was estimated by the dilution plate method using NA medium. In addition, 5 g of each substrate sample was dried at 80°C until a constant weight was reached. The dry weight (DW) of each substrate was recorded, and the number of culturable bacterial CFUs in each gram of substrate was calculated (CFU/g DW).

### PCR amplification of *16S rRNA* for illumina sequencing

During each irrigation cycle, 1 L of each influent and effluent nutrient solution sample was filtered through a 0.22-μm filter (Millipore Corp., Bedford, MA). The filter membrane was then cut using a pair of sterile scissors, and the genomic DNA was extracted using an E.Z.N.A.^®^ Water DNA kit (OMEGA, Georgia, USA) according to the manufacturer’s instructions. Genomic DNA from the substrate samples was extracted using a Fast DNA^™^ Spin Kit for Soil (MP Bio, CA, USA). The extracted genomic DNA was purified using a TIANquick Midi Purification kit (TIANGEN, Beijing), and the DNA concentration was measured using a NanoDrop^TM^ One instrument (Thermo Scientific, MA, USA). DNA integrity was examined via 1% agarose gel electrophoresis.

From the extracted genomic DNA samples, the V3-V4 region of the *16S rRNA* gene was amplified with the primer pair 338F (5'-ACTCCTACGGGAGGCAGCAG-3')/806R (5'-GGACTACHVGGGTWTCTAAT-3'), which contained the barcode sequences for a Multiplex Identifier tag. PCR amplification was performed with an initial denaturation at 95°C for 5 min, followed by 32 cycles of denaturation at 95°C for 45 s, annealing at 55°C for 50 s, and elongation at 72°C for 45 s, with a final incubation at 72°C for 10 min. The amplifications were confirmed by electrophoresis on a 2% agarose gel. Next, PCR amplicons were purified individually with a QIAquick Gel Extraction Kit (QIAGEN, Hilden, Germany) and then pooled together in equal amounts. The sequencing library was generated using an Ion Plus Fragment Library Kit (Thermo Fisher Scientific, USA) following the manufacturer’s instructions and quantified with an Agilent Bioanalyzer 2100 system. Illumina sequencing was performed by Allwegene Technology Co., Ltd. (Beijing, China) using a paired-end method with an Illumina MiSeq PE300 platform. The sequencing data have been deposited in the NCBI Sequence Read Archive (PRJNA576116).

### Sequencing data analysis

Raw data generated from the high-throughput sequencing run were processed and analyzed following the pipelines of Mothur (v1.31.2) and QIIME (v1.8.0) [25,26]. Sequence reads were trimmed so that the average Phred quality score for each read was above 20. After trimming, the reads were assembled using Uparse software (v7.0.1001) [27], and reads that could not be assembled were discarded. Chimera sequences were identified and removed using UCHIME (v4.2) [27]. Quality sequences were subsequently assigned to samples according to their unique barcode, and sequence clustering was performed using the UPARSE pipeline with a similarity cutoff of 97% [28], after which samples were clustered into operational taxonomic units (OTUs) [29]. Taxonomies were assigned to each OTU using the RDP Naïve Bayesian Classifier with custom reference databases. OTUs with RDP classifications that did not match the bacterium kingdom were removed.

The relative abundance (%) of individual taxa within each community was estimated by comparing the number of sequences assigned to a specific taxon to the number of total sequences obtained for that sample. The community structure was statistically analyzed at the phylum, genus and species classification levels. The top10 phyla and top30 genera, as well as the known bacterial species with maximum relative abundance ≥1% were listed. Rarefaction analysis was performed by Mothur. The intrasample rarefaction curves were generated using a resampling without a replacement approach [30]. Alpha diversity analysis, which included the Shannon, Simpson, and Chao1 indexes, was performed using the summary single command of Mothur software (v1.31.2) [31]. Distances were calculated using the “vegdist” function of the package “vegan” for Bray-Curtis distances. Nonmetric multidimensional scaling (NMDS) was performed using the “vegan” package [32]. The major microbial player analyses and graphics were performed using the average relative abundance and relative frequency of each OTU in three replicates.

### Inoculation of cucumber seedlings with effluent microbes

Two liters of effluent nutrient solution samples from the first, third and sixth irrigation cycles were filtered through a 0.22-μm filter, and the filter membrane was then cut into 0.5×0.5 cm pieces. After incubating the filter pieces in 20 mL of sterile water at 28°C with shaking at 160 rpm for 20 min, the inoculation was delivered to the 7 d-old cucumber seedlings via syringes. Syringes were positioned in the potting substrate and approximately 1 cm in horizontal distance from the hypocotyl of the cucumber plant. The seedlings treated with inoculum from the initial nutrient solution were used as the control. The growth conditions for cucumber seedlings were as follows: 12-h photoperiod, 28°C/18°C day/night temperature, a relative humidity of 75%-85% and a light intensity of 300 μmol/m^2^/s. After 20 days of inoculation, the plant height and shoot and root fresh weight of cucumber seedlings were recorded, and the chlorophyll content was measured with a portable chlorophyll meter (SPAD‐502, Minolta, Japan). For each seedling, the first leaf was selected to measure the chlorophyll content, and five readings were obtained for each third leaf, avoiding main veins during measurements. The seedlings were also examined microscopically to monitor disease occurrence. Four types symptoms of bacterial diseases, namely, vascular wilt, necrosis, soft rot, and tumours, were the mainly focus. During the cultivation of seedlings, neither antibacterial reagents nor other disease control methods were applied. Three biological replicates were conducted per sample, with 10 seedlings per replicate.

### Statistical analysis

All of the data are expressed as the mean ± SD. Statistical analysis was carried out using the software package SPSS (version 21.0, Chicago, USA). Paired Student’s *t*-tests were calculated for all pairwise comparisons, and *P* values were adjusted using the FDR correction for multiple testing.

## Results

### Dynamic changes in the number of culturable bacteria in the recirculating nutrient solution and substrate samples

**Fig 1:**
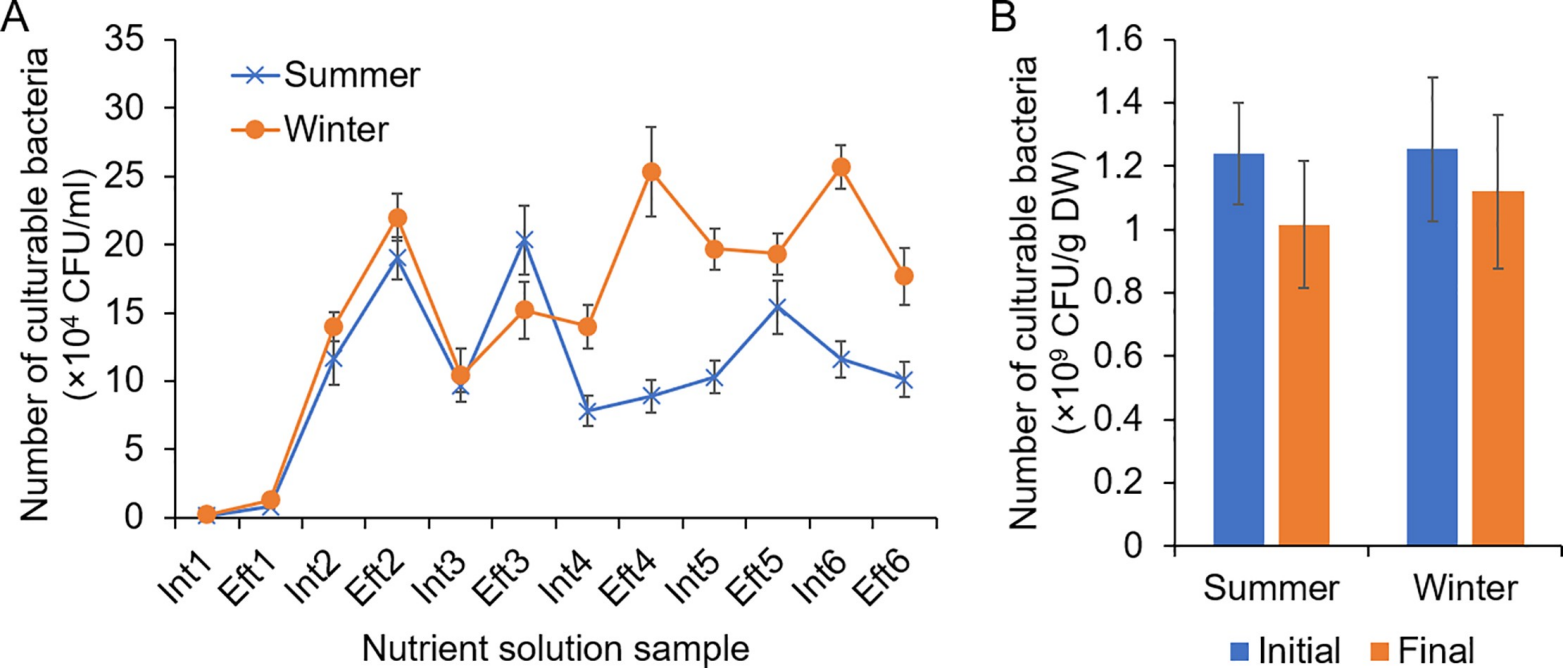
Changes in the number of culturable bacteria in the recirculating nutrient solution **(A)** and substrate **(B)** of cucumber plug seedlings cultivated under an ebb-and-flow system in summer and winter cultivation seasons. Int and Eft represent the influent and effluent nutrient solutions, respectively.

In both summer and winter, the number of culturable bacteria in the nutrient solution increased with recirculating irrigation and seedling growth (Fig 1A). At the beginning, the numbers of culturable bacteria were much lower (0.16×10^4^ and 0.25×10^4^ CFU/ml in summer and winter, respectively), while the numbers increased to approximately 1.0×10^4^ CFU/ml after the first irrigation. Subsequently, the number of culturable bacteria continued to increase and then was maintained at a relatively stable level. At the end of irrigation, the number of culturable bacteria in the nutrient solution exceeded 10^5^ CFU/ml (Fig 1A). In the substrate, the number of culturable bacteria showed no significant difference between the samples before and after seedling cultivation in both summer and winter (Fig 1B).

### Data analysis by illumina sequencing

Using Illumina sequencing of the bacterial 16S rRNA amplicons from 36 nutrient solution samples (six influent and six effluent nutrient solution samples with three replicates) collected in summer, we obtained 1 842 929 raw reads. From the six substrate samples, 537 152 raw reads were obtained. From those raw reads of the nutrient solution and substrate samples, 1 533 012 and 457 158 clean reads, respectively, were obtained after the low-quality sequences and errors were omitted. After subsampling to the minimum read by BLAST and taxonomic assignment, 2 203 and 1 040 OTUs were obtained in the nutrient solution and substrate samples, respectively (S1 Table). For the samples collected in winter, 2 006 305 and 420 184 raw reads were obtained from the 36 nutrient solution samples and six substrate samples, respectively. From these data, 1 841 398 and 345 522 clean reads were obtained. After subsampling and taxonomic assignment, 2 706 and 1 479 OTUs were obtained in the nutrient solution and substrate samples, respectively (S1 Table). At this sequencing depth, both the rarefaction curves and Shannon-Wiener curves began to level off, suggesting that the bacterial communities were reasonably well characterized with our sampling effort (S4 Fig).

### Dynamic changes in the bacterial diversity in recirculating nutrient solution and substrate samples

**Fig 2:**
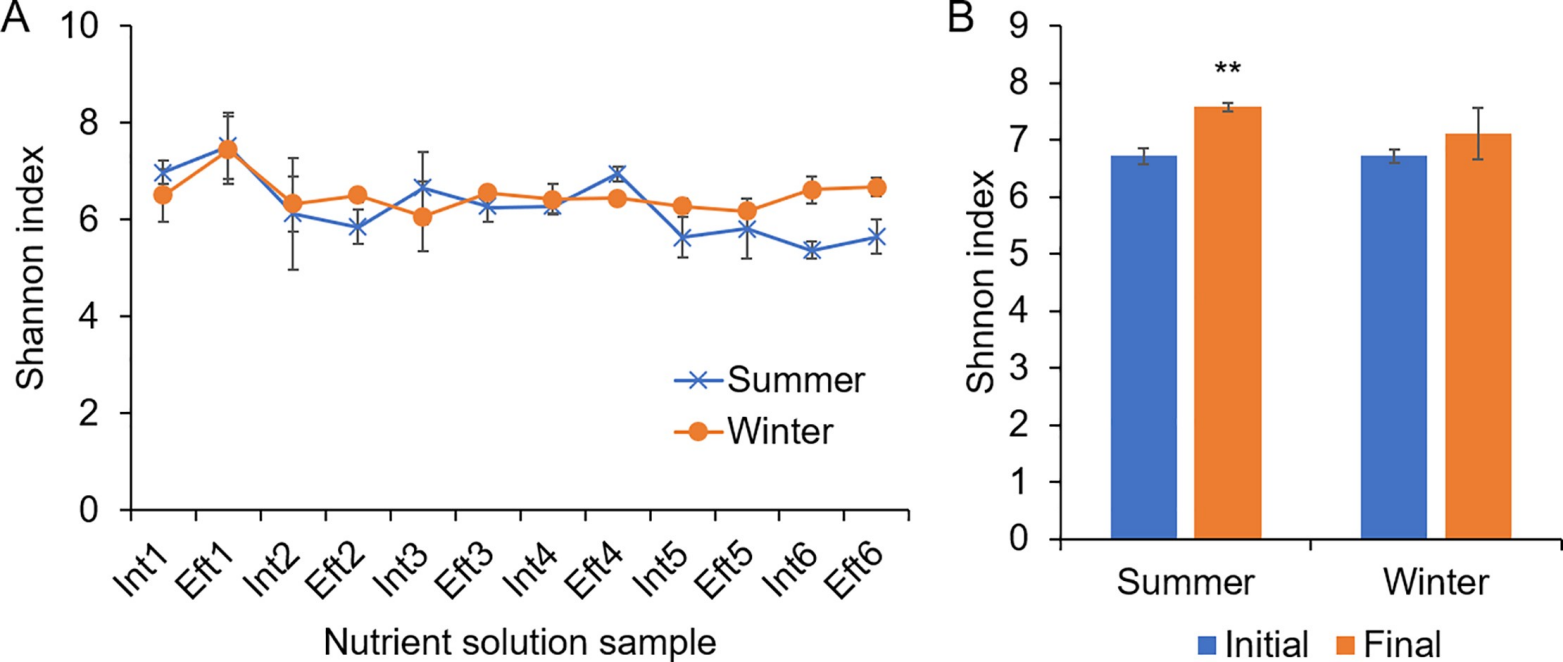
Dynamic changes in the Shannon index of bacterial communities in the recirculating nutrient solution **(A)** and substrate **(B)** samples from the ebb-and-flow system for cucumber plug seedling cultivation. **, *P* < 0.01 compared to the corresponding initial values.

The Shannon index was used to assess the microbial diversity in the recirculating nutrient solution and substrate samples. For the nutrient solution samples, in both summer and winter, the Shannon index increased after the first irrigation cycle, and subsequently, the values decreased with recirculating irrigation (Fig 2A). For the substrate samples, the Shannon index increased significantly after seedling cultivation in summer but showed no significant change in winter (*P* > 0.05, Fig 2B).

Furthermore, the overall Shannon indexes of bacterial communities between the influent and effluent nutrient solution sample groups were compared (S5 Fig). In summer, the Shannon index showed no significant difference (*P* = 0.232), while in winter, the Shannon index in the effluent sample group was slightly higher than that in the influent group (*P* < 0.05).

### Comparison of the bacterial compositions between the recirculating nutrient solution and substrate sample groups

**Fig 3:**
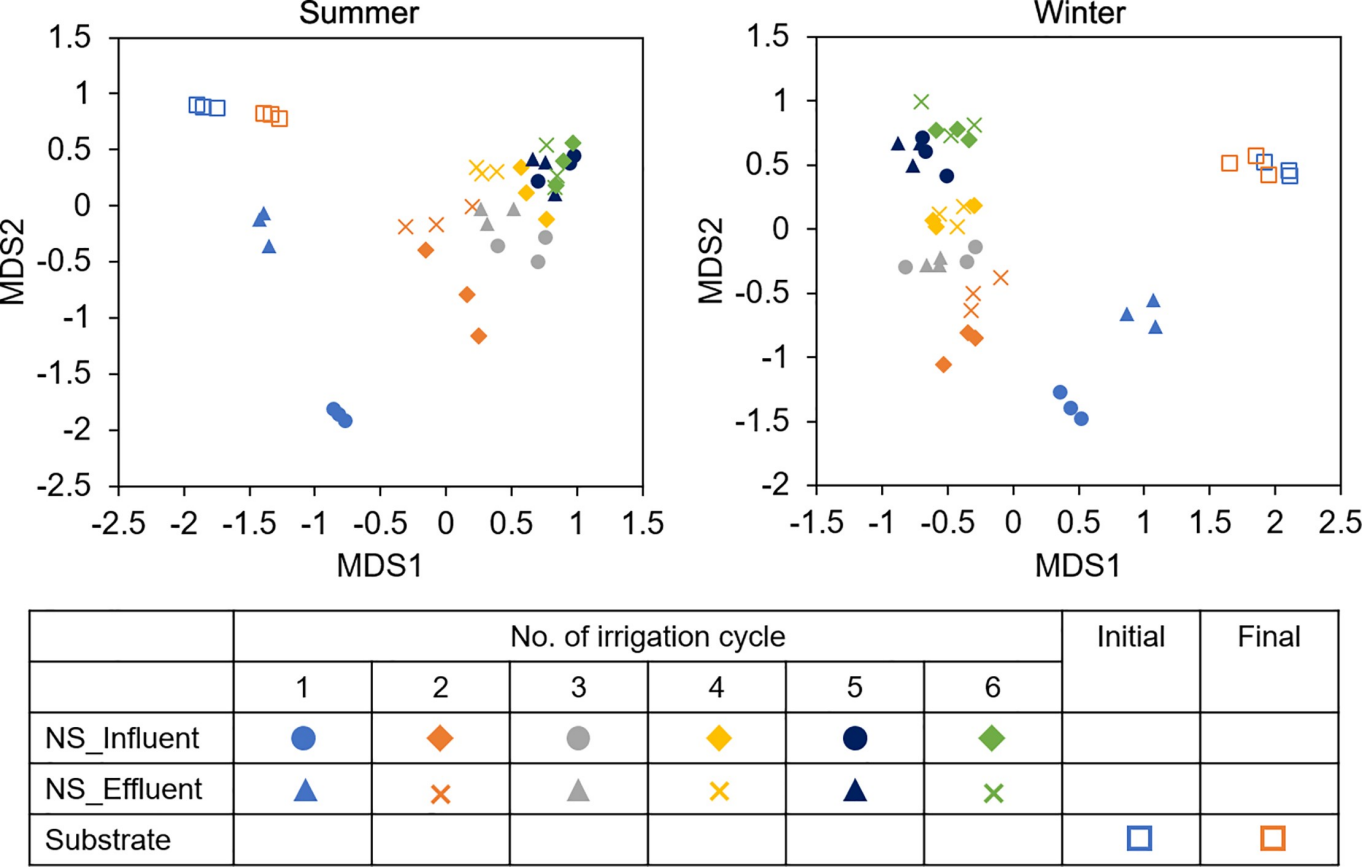
NMDS plots for Bray-Curtis distances of bacterial communities in the recirculating nutrient solution and substrate of cucumber plug seedlings cultivated in an ebb-and-flow system.

**Fig 4:**
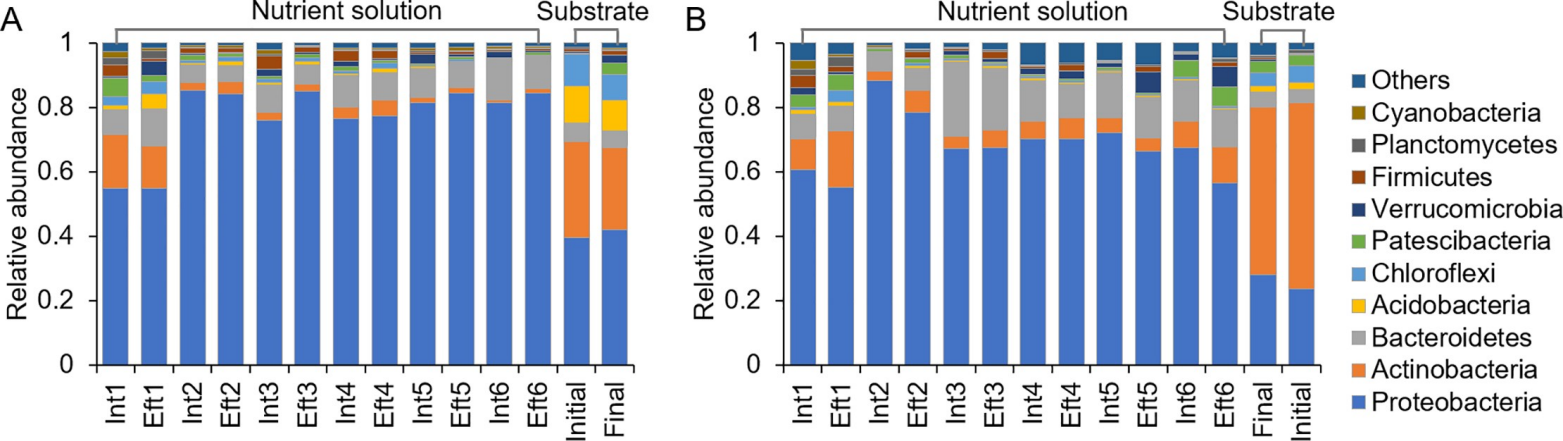
Distribution of bacterial communities at the phylum level in the recirculating nutrient solution and substrate samples of cucumber plug seedlings cultivated in an ebb-and-flow system in summer **(A)** and winter **(B).** Int and Eft represent the influent and effluent nutrient solution samples, respectively.

The similarity of the bacteria in the recirculating nutrient solution and substrate samples was analyzed using NMDS analysis based on the Bray-Curtis distance (Fig 4). The NMDS plots revealed that the nutrient solution samples varied considerably, while the substrate samples clustered more closely together. In the case of the nutrient solution samples, the distance between the influent and effluent samples in the same irrigation cycle was close, indicating a high similarity of the bacterial communities in these samples. In addition, the NMDS plot showed a clear progression of the bacterial communities over time (Fig 3). A clear distinction between the solution samples of the first irrigation cycle (Int1 and Eft1) and other nutrient solution samples was revealed. The Int1 sample was clustered more closely to the substrate group than the Eft1 sample was. With recirculating irrigation, the distances between the influent and effluent nutrient solutions gradually decreased.

### Dynamic changes in the bacterial composition in the recirculating nutrient solution and substrate samples

In the recirculating nutrient solutions, the predominant bacterial phyla included Proteobacteria, Bacteroidetes, and Actinobacteria in both summer and winter (Fig 4). Proteobacteria was the phylum with the highest relative abundance, and its relative abundance increased after the first irrigation, while the relative abundance of Actinobacteria decreased. In addition to Proteobacteria, Actinobacteria, and Bacteroidetes, the predominant phyla included Acidobacteria, Patescibacteria, Firmicutes, Chloroflexi, Verrucomicrobia, Planctomycetes and Cyanobacteria (Fig 4). The composition of the bacterial community in the substrate samples was quite different from that observed in the nutrient solution samples. Actinobacteria was the most abundant phylum in the substrate, followed by Proteobacteria. The other predominant phyla also included Chloroflexi, Acidobacteria, and Bacteroidetes. Their relative abundances in the substrate showed no significant change after seedling cultivation.

**Fig 5:**
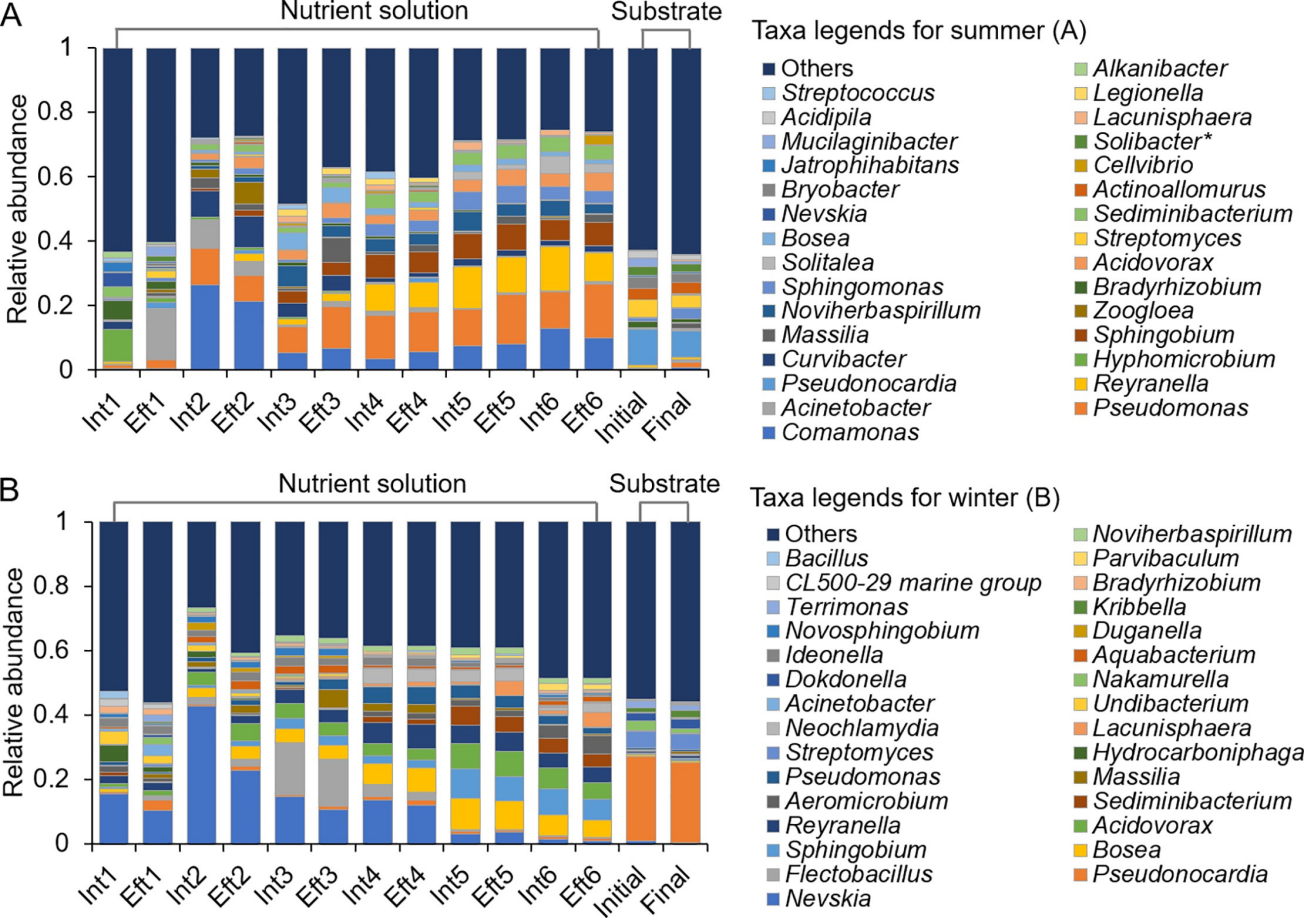
Distribution of bacterial communities at the genus level in the recirculating nutrient solution and substrate samples of cucumber plug seedlings cultivated in an ebb-and-flow system in the summer **(A)** and winter **(B)** cultivation seasons. The y-axis shows the relative abundances of the top 30 genera. Asterisks in the legend indicate taxonomic bins containing OTUs that could not be resolved to the genus level. Int and Eft represent the influent and effluent nutrient solution samples, respectively.

At the genus level, the bacterial communities in the nutrient solutions were quite different in summer and winter (Fig 5). In summer, the predominant genera included *Comamonas*, *Pseudomonas*, *Acinetobacter*, *Reyranella*, *Hyphomicrobium*, *Sphingobium*, *Massilia*, *Bradyrhizobium*, *Sphingomonas*, and *Acidovorax* (Fig 5A). With recirculating irrigation, the relative abundance of *Bradyrhizobium* gradually decreased, whereas the abundances of *Pseudomonas*, *Reyranella*, *Sphingobium*, *Sphingomonas*, and *Acidovorax* gradually increased. At the end of irrigation, *Pseudomonas* became the most predominant genus in the nutrient solution, and its relative abundance reached 16.81% (Fig 5A). For *Comamonas*, the relative abundance increased markedly during the second irrigation cycle; then, its relative abundance gradually decreased but remained higher than that in the Int1 sample. Similarly, the relative abundance of *Acinetobacter* in the In1 sample was only 0.25%, but it increased after the first irrigation (Eft1 sample, 15.77%) and then gradually decreased to the initial level. The genus *Massilia* was only predominant in the effluent samples (Fig 5A). In winter, the bacterial communities in the recirculating nutrient solution were mainly composed of *Nevskia*, *Bosea*, *Sphingobium*, *Acidovorax*, *Pseudomonas*, and *Hydrocarboniphaga* (Fig 5B). With recirculating irrigation, the relative abundances of *Bosea*, *Sphingobium*, and *Acidovorax* increased gradually, whereas *Nevskia* and *Hydrocarboniphaga* decreased overall.

For the substrate samples collected both in summer and winter, the predominant bacterial genera included *Pseudonocardia* and *Streptomyces*. Additionally, the genera *Sphingomonas*, *Actinoallomurus*, *Bryobacter*, *Bradyrhizobium*, *Mucilaginibacter*, *Massilia*, and *Acidipila* more commonly occurred in summer, and *Nakamurella*, *Dokdonella*, *Kribbella*, and *Terrimonas* showed a predominant distribution in winter (Fig 5). In summer, the relative abundance of *Massilia* and *Sphingomonas* showed a more than 2-fold increase in the final substrate samples at the end of the seedling period, while that of *Mucilaginibacter* showed a decreasing pattern (Fig 5A). In winter, the relative abundances of *Nakamurella* and *Terrimonas* decreased, and the relative abundance of *Kribbella* increased (Fig 5B). It should also be noted that the bacterial genera that changed in the recirculating nutrient solutions did not significantly increase or decrease in the substrate samples.

**Fig 6:**
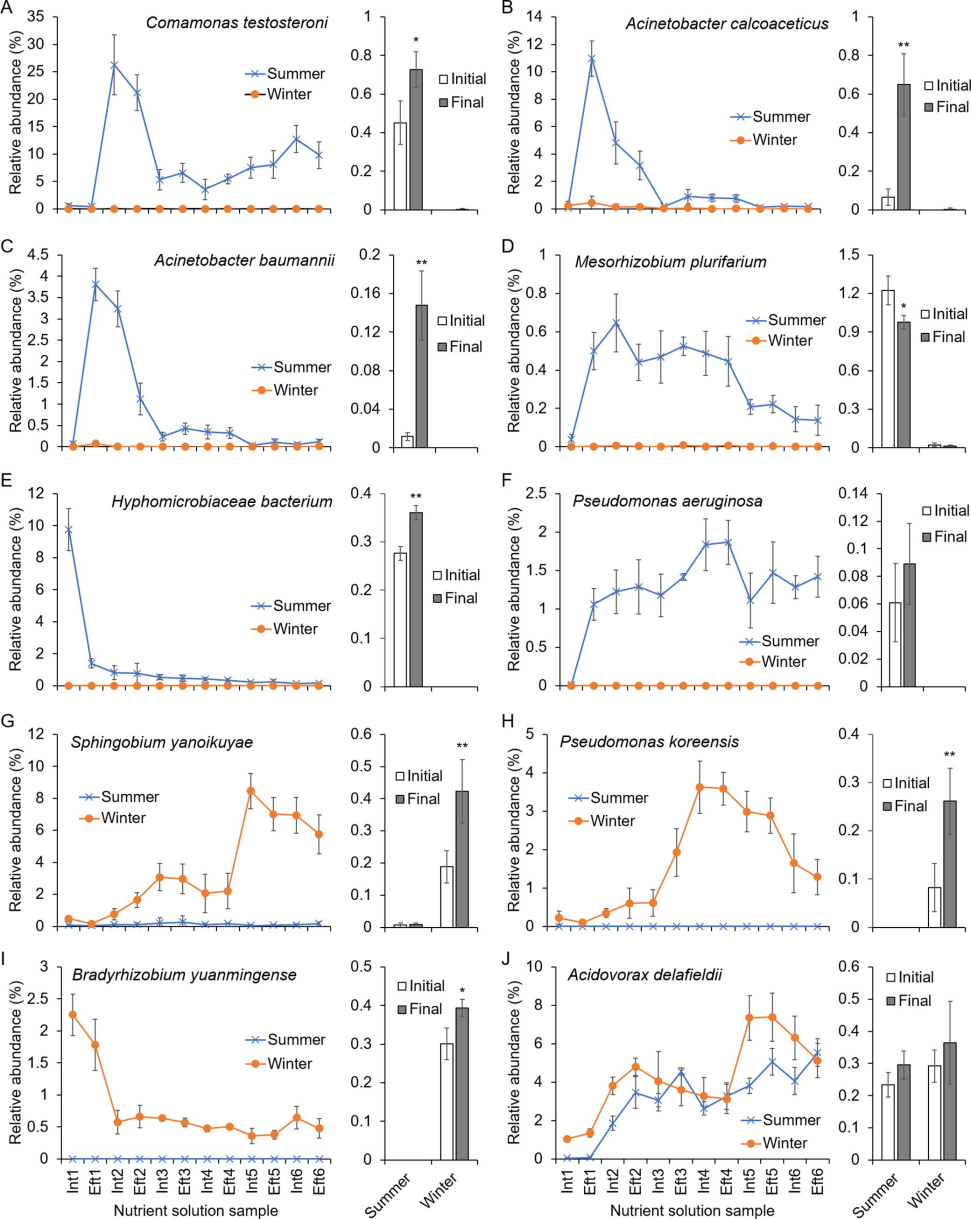
Dynamic changes in ten predominant bacterial species in the recirculating nutrient solution (lines) and substrate (bars) samples of cucumber plug seedlings cultivated in an ebb-and-flow system in the summer and winter cultivation seasons. The bacterial species belonging to the known species and showing relative abundance ≥1% in at least one sample are shown. Int and Eft represent the influent and effluent nutrient solution samples, respectively. *, *P* < 0.05 and **, *P* < 0.01 compared to the corresponding initial values.

Some OTUs were assigned to the species level. Ten bacteria with relative abundance ≥1% in at least one sample belonged to the known classified species (Fig 6). In summer, *Comamonas testosteroni* showed a low distribution (approximately 0.5%) in the nutrient solution at the beginning of irrigation. After the second irrigation cycle, the relative abundance of *C*. *testosteroni* increased immediately to more than 20%, and after that, its relative abundance gradually decreased with recirculating irrigation (Fig 6A). In the substrate samples, *C*. *testosteroni* was also present only in summer, with its relative abundance increasing slightly (1.61-fold) after seedling cultivation (Fig 6A). A similar trend of first rising and then decreasing was also found in two bacteria of the *Acinetobacter* genus, *A*. *calcoaceticus* and *A*. *baumannii*. Both populations increased markedly after the first irrigation cycle and then decreased. Until the third irrigation cycle, their relative abundances had been reduced to trace levels. In substrate samples, both *A*. *calcoaceticus* and *A*. *baumannii* were also present only in summer, and their relative abundances increased significantly after cucumber seedling cultivation (*P* < 0.01; Fig 6B and 6C). A similar changing pattern in the nutrient solution was also found for *Mesorhizobium plurifarium*, but its relative abundance decreased in the final substrate samples (Fig 6D). In summer, the predominant bacteria also included *Hyphomicrobiaceae bacterium* and *Pseudomonas aeruginosa*; the former was only present predominantly in the Int1 sample (Fig 6E), while the latter accumulated gradually during irrigation (Fig 6F). The relative abundances of all the above bacteria were very low in the solution and substrate samples collected in winter. In contrast, *Sphingobium yanoikuyae*, *Pseudomonas koreensis*, *Bradyrhizobium yuanmingense* could accumulate only in winter in the recirculating nutrient solution, but with different changing patterns (Fig 6G, 6H and 6I). The relative abundance of *S*. *yanoikuyae* increased gradually in the recirculating nutrient solution, and by the end of irrigation, *S*. *yanoikuyae* became the most abundant bacterium (Fig 6G). The relative abundance of *P*. *koreensis* increased gradually until the fourth irrigation cycle and then decreased with irrigation (Fig 6H). The relative abundance of *B*. *yuanmingense* decreased immediately in the first irrigation cycle (Fig 6I). In the substrate, *S*. *yanoikuyae*, *P*. *koreensis*, and *B*. *yuanmingense* also showed a specific distribution in winter, and their relative abundances increased significantly after seedling cultivation (Fig 6G, 6H and 6I). *Acidovorax delafieldii* accumulated gradually in the recirculating nutrient solution in both summer and winter. However, in the substrate, no significant change was observed between the initial and final samples (*P* > 0.05; Fig 6J).

### Promotion effects of effluent microbes on cucumber seedling growth

The use of inoculum isolated from the Eft3 and Eft6 samples had a plant growth-promoting effect on the cucumber seedlings, obtaining higher plant heights, fresh weight and chlorophyll contents than those of seedlings group grown when the inoculum used was Eft1 and the control group. There was no significant difference between the latter two groups (Table 2). Furthermore, none of the cucumber seedlings showed any detectable disease symptoms, including vascular wilt, necrosis, soft rot, and tumours (data not shown). These results suggested the presence of plant growth-promoting microbes but the absence of the plant pathogens in the effluent nutrient solutions in the recirculating ebb-and-flow system of cucumber plug seedlings.

**Table 2:** Effects of inoculation with effluent microbes on fresh weight and chlorophyll content of cucumber seedlings.

Inoculation	Plant height (cm)	Shoot fresh weight (g)	Root fresh weight (g)	Chlorophyll content (SPAD reading)
Control	9.85 ± 0.62 b	2.23 ± 0.06 b	0.98 ± 0.11 b	37.72 ± 1.26 b
Eft1	9.57 ± 0.88 b	2.38 ± 0.11 b	1.02 ± 0.05 b	36.85 ± 2.31 b
Eft3	10.20 ± 0.76 ab	2.98 ± 0.22 a	1.25 ± 0.12 ab	40.87 ± 1.99 a
Eft6	11.37 ± 0.45 a	2.83 ± 0.25 a	1.27 ± 0.07 a	42.33 ± 2.24 a

Different letters indicate the significant differences among various treatments at *P* < 0.05.

## Discussion

In this study, the dynamic changes in bacterial communities in an ebb-and-flow subirrigation system were investigated. Illumina sequencing of the V3-V4 region of *16S rRNA* genes and metagenomic library analysis enabled us to observe the global bacterial community structure in the recirculating nutrient solution and substrate for the cucumber plug seedlings.

Both bacterial number and diversity increased immediately after the first irrigation cycle (Figs 1 and 2), suggesting that the indigenous bacteria in the substrate might be the major resource of the bacteria in the nutrient solution. The bacterial propagules could be leached from the substrate in plug trays and transported by the recirculated irrigation water [4]. This speculation was also consistent with the findings in the NMDS analysis, which revealed that Eft1 samples were clustered more closely to the substrate group than the Int1 samples (Fig 3). In the recirculating ebb-and-flow system, the bacterial communities in the influent and effluent samples in each irrigation cycle tended to be similar. Additionally, during the late irrigation cycles, the samples were more similar than those in the early irrigation cycles.

The bacterial communities in the nutrient solution were further analyzed (Figs 4–6). Proteobacteria was the most abundant phylum but comprised different genera in summer and winter. In summer, Proteobacteria comprised *Comamonas*, *Pseudomonas*, *Bradyrhizobium*, and *Acinetobacter*, while in winter, Proteobacteria included *Nevskia*, *Sphingobium*, and *Acidovorax*. These results were partially consistent with the previous findings, which reported that *Pseudomonas* was the most predominant bacterial genus and accounted for more than 40% of the total cultivable bacterial flora in the recycled nutrient solution samples in a soilless system [33,34]. The relative abundances of *Comamonas* and *Pseudomonas* in summer and *Sphingobium* and *Acidovorax* in winter increased with recirculating irrigation. This observation might be explained by the fact that the temperature, high nutrient content and dissolved oxygen concentration of the nutrient solutions offer an optimal growth environment for these genera [34]. The seasonal variations in the bacterial populations might be due to the different indigenous bacterial compositions in the irrigation water. Seasonal bacterial community dynamics in the water have been reported previously, with temperature being the most important variable correlating with bacterial community composition changes [35–37]. Additionally, the different environmental factors in the greenhouse and physiochemical parameters of nutrient solutions were the other explanation for the distinct change patterns between summer and winter (S1 and S2 Figs).

Furthermore, some dominant OTUs could be assigned to the known species (Fig 6), and seven of these bacterial species of them were reported previously to have some known function. For example, *C*. *testosteroni* is the most commonly used biofilm bacterium in sewage treatment because it can degrade aromatic substances in water, such as polycyclic aromatic hydrocarbons, indoles, and nonylphenols [38,39]. In this study, the relative abundance of *C*. *testosteroni* showed an overall increase in both nutrient solution and substrate, but this presence was only in summer. In summer, two *Acinetobacter* bacteria, *A*. *baumannii* and *A*. *calcoaceticus*, were detected transiently in the nutrient solution during the early stage of irrigation. These two bacteria are grouped into the ACB complex, which comprises opportunistic nosocomial pathogens and one of the most important multidrug-resistant bacteria in hospitals worldwide [40,41]. The presence of these ACB complex bacteria is not beneficial for the workers. A similar finding was also observed for *P*. *aeruginosa*, a major human opportunistic pathogen that causes numerous acute and chronic infections [42]. Preventing these human pathogenic bacteria from entering the greenhouse would be critical for workers. *A*. *baumannii* and *A*. *calcoaceticus* were further enriched in the substrate after seedling cultivation. It has been reported that *A*. *baumannii* has IAA productive capability, and the produced IAA can enhance root growth in *A*. *baumannii* and kidney bean plant cocultures [43]. For *P*. *aeruginosa*, moisture and high humidity are necessary for its colonization and survival, but this species is not a typical plant pathogen, and it does not produce pectic enzymes normally associated with pathogens that cause rot [44]. In contrast, *P*. *aeruginosa* can produce the antibiotic 2,4-diacetylphloroglucinol and has the potential to suppress *Fusarium* root and stem rot diseases in greenhouse cucumbers [45]. In winter, *P*. *koreensis* and *S*. *yanoikuyae* accumulated in both nutrient solution and substrate. *P*. *koreensis* was identified as an agricultural soil-borne bacterium, and many strains worldwide have been identified with diverse capacities ranging from nitrogen fixation [46] to the production of biosurfactant compounds and suppression of plant diseases caused by oomycete pathogens and *Pythium* species [47,48]. *S*. *yanoikuyae* is often used in environmental pollution control, with its ability to degrade polycyclic aromatic hydrocarbons (PAHs) in PAH-contaminated soils [49]. Another PAH-degrading bacterium, *A*. *delafieldii*, was found to accumulate in the recirculating nutrient solution in both summer and winter. *A*. *delafieldii* is a nutritionally versatile species frequently present in wastewater treatment plants [50–52]. The successful colonization of *S*. *yanoikuyae* and *A*. *delafieldii* in the nutrient solution would provide some potential candidates for the self-purification of the nutrient solution in recirculating ebb-and-flow systems. Of course, the effects of these PAH-degrading bacteria on plant growth, especially in cucumber seedling growth, also need further experimental investigation.

The substrate samples possessed bacterial compositions that were quite different from those of the nutrient solution. Two dominant genera, *Pseudonocardia* and *Streptomyces*, were present in both summer and winter (Fig 5). Additionally, at the species level, most of the dominant bacteria in the nutrient solution accumulated in the substrate after seedling cultivation (Fig 6). When delivering the nutrient solution samples to the cucumber seedlings, seedling growth was significantly promoted (Table 2). These findings suggested that the potential plant growth-promoting bacterial candidates identified in this study could be applied in ebb-and-flow systems through inoculation in the nutrient solution.

In this study, there was no presence of any disease on the cucumber plug seedlings. Pathogenic bacteria were also negative in all nutrient solution and substrate samples. None of the common bacterial pathogens of cucumber, such as *Xanthomonas campestris* pv. cucurbitae (causing bacterial leaf spot) [53], *Pseudomonas syringae* pv. lachrymans (causing bacterial angular leaf spot pathogen) [54], *Erwinia tracheiphila* (causing bacterial wilt) [55] and *Pectobacterium carotovorum* subsp. brasiliense (causing bacterial soft rot) [56], was detected in this ebb-and-flow system. Additionally, inoculation of the cucumber seedlings with microbes isolated from the effluent solution samples did not lead to any disease (data not shown). Our results suggested the absence of plant pathogenic bacteria in this recirculating ebb-and-flow system. Many previous reports also found a negative incidence of bacterial pathogens in subirrigation systems [5,7,57]. In a closed system, good sanitation and the use of appropriate control methods are necessary to ensure the proper sanitary conditions for plant cultivation. Maintaining the production area and providing nutrient solutions free of plant debris are simple and efficient procedures to avoid diseases in recirculating systems [7]. Without disinfection, the native bacteria could survive in the nutrient solution and play roles in plant growth promotion and self-purification of irrigation water. Similarly, Sanogo and Moorman reported that stem base necrosis symptoms and damping-off in cucumber plants occurred only in pathogen-inoculated treatments [58]. Certainly, in our further studies, the dynamic microbial communities should be tested in an ebb-and-flow system where a plant pathogen is actually added. Additionally, the pathogens on cucumber are often of fungal origin, and further analysis of the fungal communities is needed.

## Conclusions

In conclusion, the distribution of bacteria showed obvious dynamic changes in the recirculating nutrient solution and substrate in an ebb-and-flow system. The results indicated that the substrate and irrigation water might be the primary sources of bacterial transfer to the nutrient solution. Additionally, the environmental conditions could have influenced the bacterial diversity in different seasons. Furthermore, we particularly focused on the changes in certain potentially pathogenic and beneficial bacteria. The plant pathogenic bacteria were absent in all nutrient solution and substrate samples, while some beneficial bacteria, such as *C*. *testosteroni*, *P*. *aeruginosa*, and *P*. *koreensis*, colonized the nutrient solution and increased with recirculating irrigation. The absence of plant pathogens and the presence of plant growth-promoting microbes were confirmed by the further inoculation experiments. However, one concern was about the human pathogenic bacteria in the nutrient solution, including *A*. *baumannii*, *A*. *calcoaceticus*, and *P*. *aeruginosa*, indicating the importance of preventing the human pathogens from entering the greenhouse. The comprehensive characterization of the bacterial dynamics in a recirculating ebb-and-flow system will lay a foundation for further research and applications in cucumber plug seedling production.

## Supporting information

S1 Table Statistical analysis of the bacterial *16S rRNA* sequencing data in the recirculating nutrient solution and substrate samples from an ebb-and-flow system for cucumber seedling cultivation. (DOCX)

S1 Fig A schematic of an ebb-and-flow system employed to produce cucumber plug seedlings. (DOCX)

S2 Fig Dynamic changes in EC and DO content in the nutrient solution during the seedling cultivation period in summer and winter. (DOCX)

S3 Fig Daily trends of light intensity, relative humidity, air temperature, substrate temperature and solution (NS) temperature during the cultivation period in summer and winter. The curves are the means of the data at each time point during the seedling cultivation period.
(DOCX)

S4 Fig Rarefaction curves of the bacteria in the nutrient solution and substrate samples based on the OTU numbers **(A, B)** and Shannon indexes **(C, D).** Samples from A and C were collected in the summer cultivation season, and samples from B and D were collected in winter.
(DOCX)

S5 Fig Comparison of Shannon indexes of bacterial communities between the influent and effluent nutrient solution sample groups. ^*^, *P* < 0.05 compared to the corresponding values.
(DOCX)
