# Supplementary material for: Dynamic changes in bacterial communities in the recirculating nutrient solution of cucumber plug seedlings cultivated in an ebb-and-flow subirrigation system
Source: PLoS One. 2020 Apr 30;15(4):e0232446. doi: 10.1371/journal.pone.0232446 (PMC7192414; doi:10.1371/journal.pone.0232446)
Supplement: S1 Table — (DOCX) [file pone.0232446.s001.docx]

**Table S1. Statistical analysis of the bacterial *16S rRNA* sequencing data in the recirculating nutrient solution and substrate samples from an ebb-and-flow system for cucumber seedlings cultivation.**

| Sample type | Sample ID | Replicate | Season: summer | | | Season: winter | | |
| --- | --- | --- | --- | --- | --- | --- | --- | --- |
|  |  |  | Raw reads | Clean reads | OTUs^*^ | Raw reads | Clean reads | OTUs^*^ |
| Nutrient solution | Int1 | R1 | 19 803 | 18 543 | 565 | 30 606 | 28 295 | 703 |
|  |  | R2 | 44 574 | 40 046 | 742 | 35 985 | 34 039 | 803 |
|  |  | R3 | 49 398 | 46 509 | 679 | 21 470 | 20 246 | 686 |
|  | Eft1 | R1 | 34 428 | 33012 | 593 | 31 484 | 30 459 | 893 |
|  |  | R2 | 24 287 | 21179 | 984 | 76 236 | 69 889 | 1 088 |
|  |  | R3 | 70 771 | 67266 | 764 | 58 539 | 51 131 | 1 078 |
|  | Int2 | R1 | 49 025 | 44 021 | 366 | 30 627 | 28 174 | 739 |
|  |  | R2 | 61 141 | 54 438 | 374 | 35 632 | 32 158 | 739 |
|  |  | R3 | 37 781 | 35 039 | 582 | 69 004 | 65 061 | 581 |
|  | Eft2 | R1 | 48 073 | 35 025 | 538 | 50 887 | 44 150 | 1 116 |
|  |  | R2 | 80 949 | 61 770 | 485 | 55 180 | 47 487 | 1 019 |
|  |  | R3 | 66 588 | 48 111 | 588 | 44 503 | 39 831 | 987 |
|  | Int3 | R1 | 95 183 | 86 151 | 547 | 54 247 | 50 372 | 819 |
|  |  | R2 | 61 599 | 52 006 | 709 | 48 583 | 43 980 | 878 |
|  |  | R3 | 19 267 | 17 191 | 681 | 100 795 | 94 631 | 707 |
|  | Eft3 | R1 | 72 932 | 55 976 | 514 | 61 555 | 55 534 | 1 030 |
|  |  | R2 | 79 425 | 60 557 | 525 | 50 173 | 43 659 | 1 146 |
|  |  | R3 | 61 106 | 49 387 | 518 | 45 680 | 39 568 | 1 000 |
|  | Int4 | R1 | 23 813 | 21 134 | 548 | 131 997 | 122 822 | 887 |
|  |  | R2 | 36 925 | 32 103 | 544 | 58 756 | 52 932 | 833 |
|  |  | R3 | 59 295 | 55 836 | 544 | 49147 | 45 165 | 737 |
|  | Eft4 | R1 | 37 549 | 26176 | 590 | 36 445 | 34 001 | 711 |
|  |  | R2 | 40 640 | 29568 | 600 | 40 631 | 37 642 | 751 |
|  |  | R3 | 52 557 | 37422 | 631 | 44 589 | 38 888 | 810 |
|  | Int5 | R1 | 36 197 | 31 043 | 420 | 54 704 | 49 370 | 781 |
|  |  | R2 | 42 864 | 38 792 | 421 | 77 800 | 72 739 | 791 |
|  |  | R3 | 43 691 | 40 536 | 584 | 54 116 | 50 463 | 728 |
|  | Eft5 | R1 | 56 958 | 45 083 | 389 | 39 991 | 36 372 | 757 |
|  |  | R2 | 44 825 | 37 580 | 514 | 86 741 | 83 354 | 598 |
|  |  | R3 | 76 123 | 59 999 | 522 | 53 673 | 50 631 | 698 |
|  | Int6 | R1 | 27 481 | 22 877 | 389 | 67 698 | 62 304 | 844 |
|  |  | R2 | 29 421 | 24 192 | 420 | 73 108 | 67 151 | 841 |
|  |  | R3 | 25 413 | 18 850 | 360 | 69 828 | 67 359 | 658 |
|  | Eft6 | R1 | 72845 | 55 569 | 436 | 42 107 | 38 708 | 938 |
|  |  | R2 | 120 899 | 94 604 | 424 | 60 986 | 56 282 | 832 |
|  |  | R3 | 39 103 | 35 421 | 492 | 62 802 | 57 091 | 931 |
| Substrate | Initial | R1 | 93 906 | 82 355 | 683 | 75 363 | 59 734 | 1 098 |
|  |  | R2 | 100 904 | 89 215 | 632 | 75 738 | 62 556 | 1 102 |
|  |  | R3 | 87 876 | 74 021 | 690 | 74 922 | 61 740 | 1 138 |
|  | Final | R1 | 92 531 | 76 893 | 913 | 64 099 | 52 349 | 1 233 |
|  |  | R2 | 67 603 | 54 865 | 890 | 53 980 | 42 431 | 1 169 |
|  |  | R3 | 94 332 | 79 809 | 860 | 76 082 | 66 712 | 1 166 |

* OTUs were defined at the 97% similarity level.
