# Supplementary figures and images for: Dynamic changes in bacterial communities in the recirculating nutrient solution of cucumber plug seedlings cultivated in an ebb-and-flow subirrigation system
Source: PLoS One. 2020 Apr 30;15(4):e0232446. doi: 10.1371/journal.pone.0232446 (PMC7192414; doi:10.1371/journal.pone.0232446)

**Figure S1**


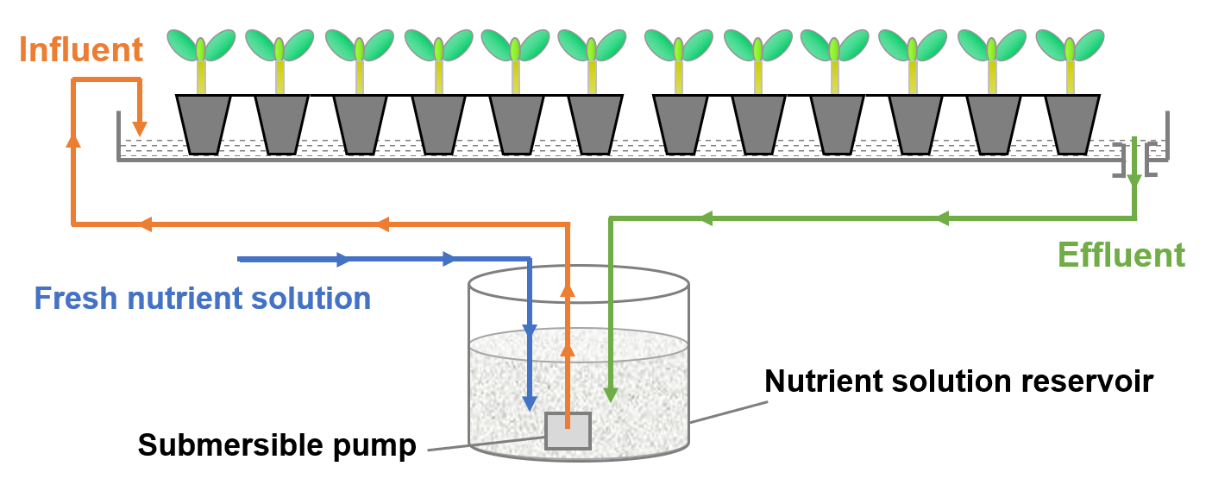


**S1 Fig. A schematic of an ebb-and-flow system employed to produce cucumber plug seedlings.**

Supplement: S1 Fig — (DOCX) [file pone.0232446.s002.docx]
