# Supplementary material for: Dynamic changes in bacterial communities in the recirculating nutrient solution of cucumber plug seedlings cultivated in an ebb-and-flow subirrigation system
Source: PLoS One. 2020 Apr 30;15(4):e0232446. doi: 10.1371/journal.pone.0232446 (PMC7192414; doi:10.1371/journal.pone.0232446)
Supplement: S2 Fig — (DOCX) [file pone.0232446.s003.docx]

**Figure S2**


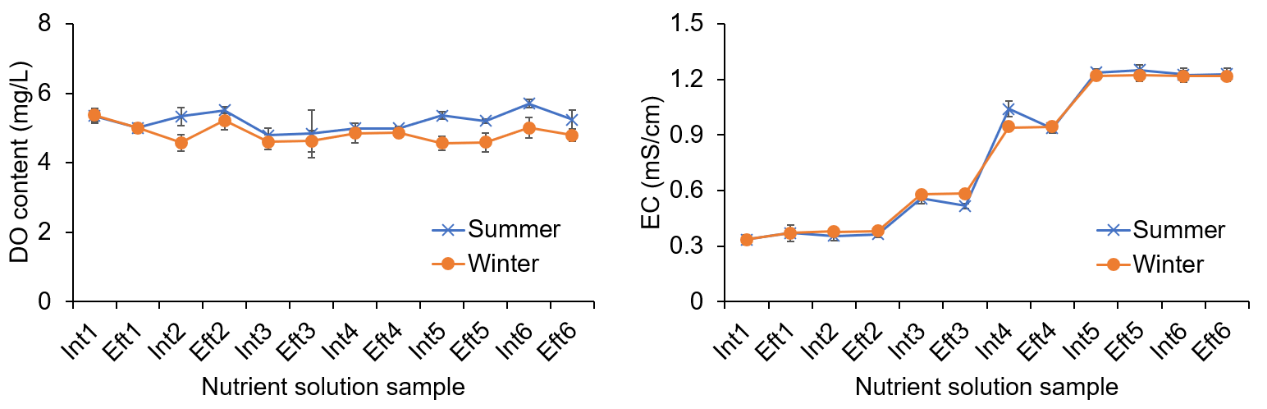


**S2 Fig. Dynamic changes in EC and DO content in the nutrient solution during the seedling cultivation period in summer and winter.**
