# Supplementary material for: Dynamic changes in bacterial communities in the recirculating nutrient solution of cucumber plug seedlings cultivated in an ebb-and-flow subirrigation system
Source: PLoS One. 2020 Apr 30;15(4):e0232446. doi: 10.1371/journal.pone.0232446 (PMC7192414; doi:10.1371/journal.pone.0232446)
Supplement: S3 Fig — The curves are the means of the data at each time point during the seedling cultivation period. (DOCX) [file pone.0232446.s004.docx]

**Figure S3**


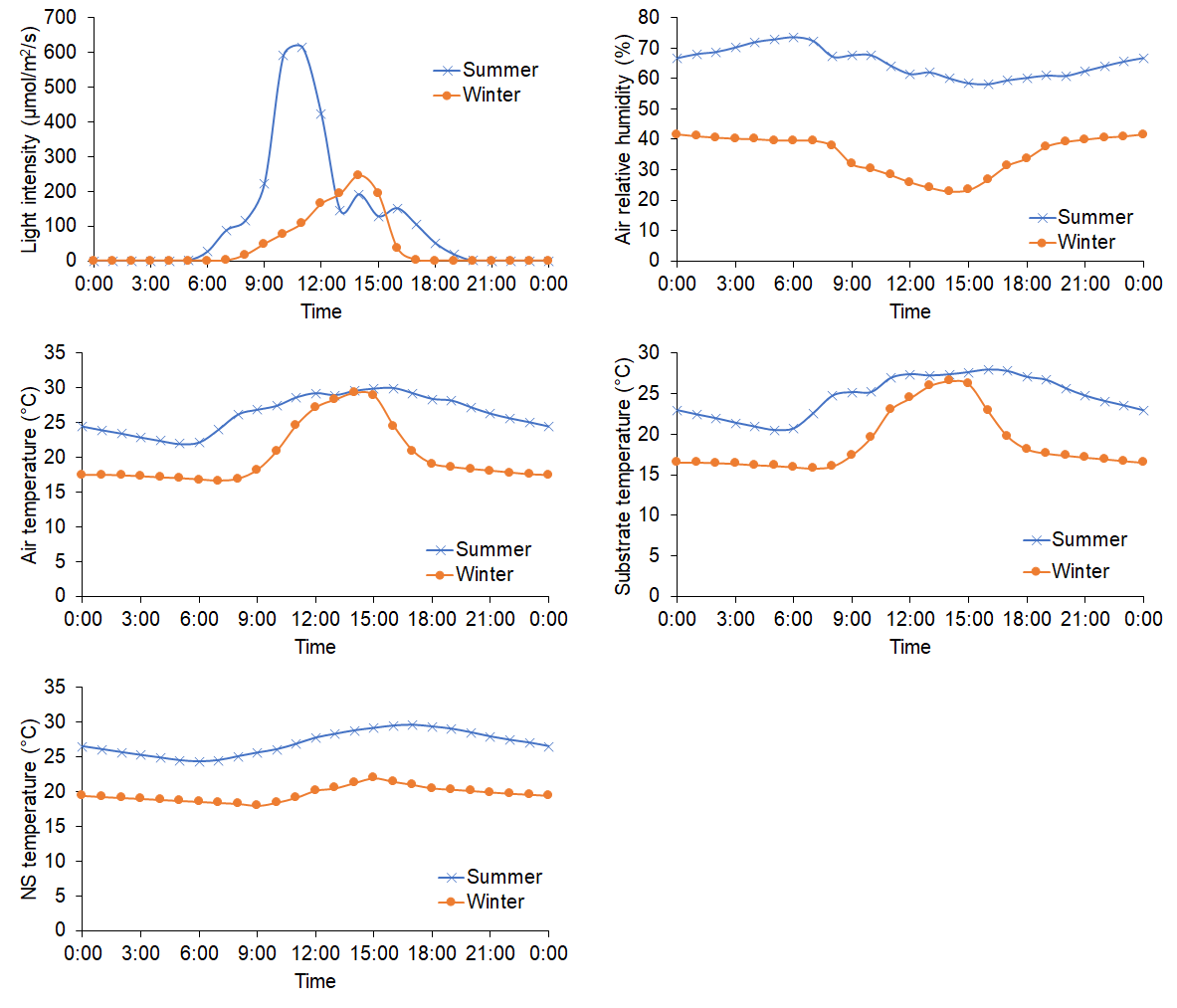


**S3 Fig. Daily trends of light intensity, relative humidity, air temperature, substrate temperature and solution (NS) temperature during the cultivation period in summer and winter.** The curves are the means of the data at each time point during the seedling cultivation period.
