# Supplementary material for: Dynamic changes in bacterial communities in the recirculating nutrient solution of cucumber plug seedlings cultivated in an ebb-and-flow subirrigation system
Source: PLoS One. 2020 Apr 30;15(4):e0232446. doi: 10.1371/journal.pone.0232446 (PMC7192414; doi:10.1371/journal.pone.0232446)
Supplement: S4 Fig — Rarefaction curves of the bacteria in the nutrient solution and substrate samples based on the OTU numbers (A, B) and Shannon indexes (C, D). Samples from A and C were collected in the summer cultivation season, and samples from B and D were collected in winter. (DOCX) [file pone.0232446.s005.docx]

**Figure S4**


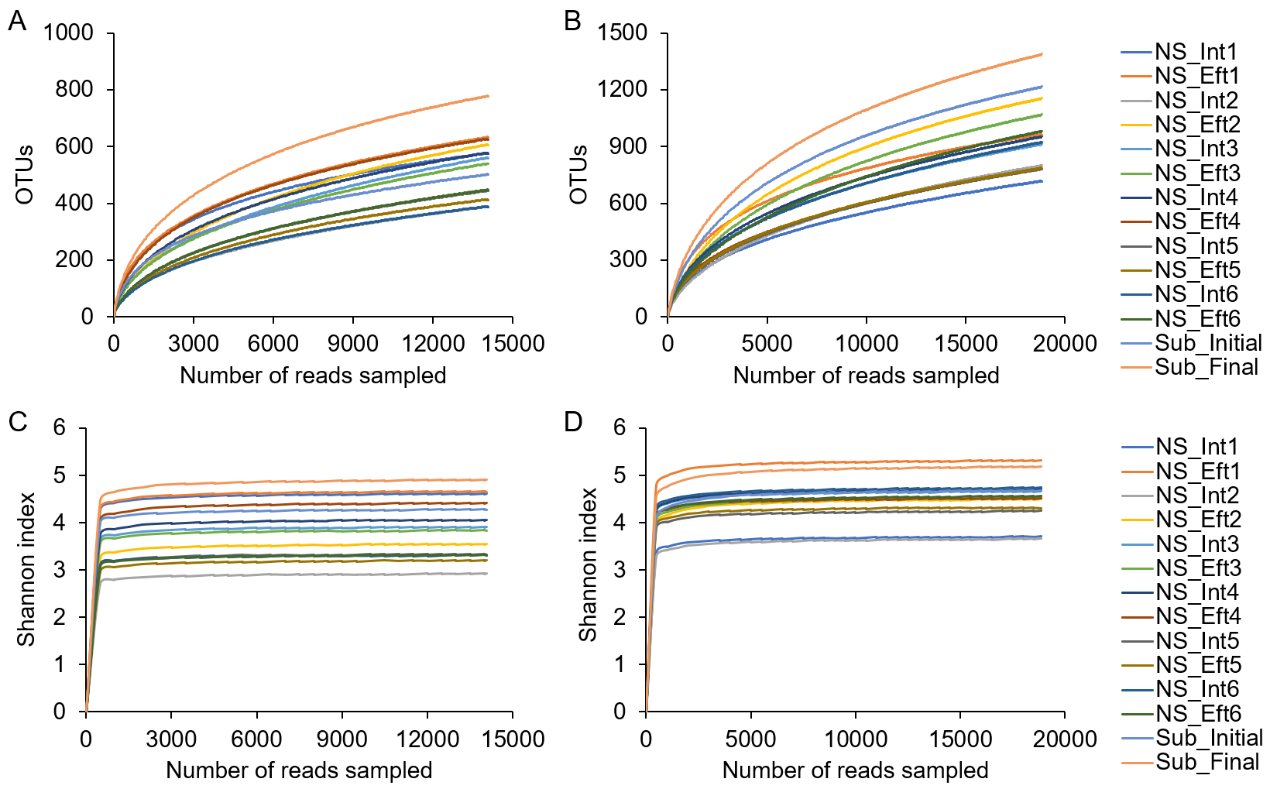


**S4 Fig. Rarefaction curves of the bacteria in the nutrient solution and substrate samples based on the OTU numbers (A, B) and Shannon indexes (C, D).** Samples from A and C were collected in the summer cultivation season, and samples from B and D were collected in winter.
