# Supplementary material for: Dynamic changes in bacterial communities in the recirculating nutrient solution of cucumber plug seedlings cultivated in an ebb-and-flow subirrigation system
Source: PLoS One. 2020 Apr 30;15(4):e0232446. doi: 10.1371/journal.pone.0232446 (PMC7192414; doi:10.1371/journal.pone.0232446)
Supplement: S5 Fig — *, P < 0.05 compared to the corresponding values. (DOCX) [file pone.0232446.s006.docx]

**Figure S5**


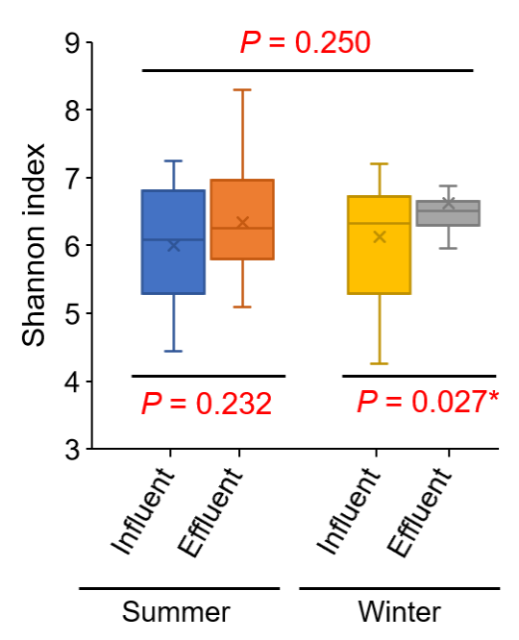


**S5 Fig. Comparison of Shannon indexes of bacterial communities between the influent and effluent nutrient solution sample groups.** ^*^, *P* < 0.05 compared to the corresponding values.
